# Physical exercise in metabolic myopathies at risk of rhabdomyolysis: a feasible approach or an unavoidable hazard?

**DOI:** 10.1007/s00421-025-05922-y

**Published:** 2025-08-04

**Authors:** Oscar Crisafulli, Daniele Diotti, Massimo Negro, Emanuela Lavaselli, Melinda Peters, Venere Quintiero, Giuseppe D’Antona

**Affiliations:** 1 CRIAMS-Sport Medicine Centre Voghera, University of Pavia, 27058 Voghera, Italy; 2 Division of Genetics and Genomics, Boston Children’s Hospital, Harvard Medical School, Boston, MA USA; 3 Department of Public Health, Experimental and Forensic Medicine, University of Pavia, 27100 Pavia, Italy

**Keywords:** Metabolic myopathies, Adapted physical exercise, Exercise physiology, Rhabdomyolysis, Glycogen storage disorders, Fatty acid oxidation disorders

## Abstract

Metabolic myopathies are a diverse group of inherited genetic disorders that disrupt carbohydrate and fatty acid metabolism, leading to impaired production of adenosine triphosphate and consequently, compromised muscle function. In many of these conditions, regardless of the specific metabolic defect, physical exercise (PE) can induce rhabdomyolysis (RML), posing a significant health risk to patients. Except for Glycogen Storage Disease (GSD) Type V, for which specific PE guidelines are available, clinical management of these conditions typically advocates for substantial physical inactivity to prevent complications. However, while this strategy helps avoid RML and its dangerous consequences, such as acute kidney failure, it also exposes patients to several long-term risks, including a decline in physical efficiency, reduced autonomy, and the emergence of comorbidities. Therefore, it is crucial to identify disease-specific PE modalities that can be safely performed to improve clinical management and enhance patients’ quality of life. The existing literature on this topic is generally limited, likely due to the rarity of these conditions. Nevertheless, a comprehensive analysis of the available evidence could provide a foundation for identifying feasible PE modalities and developing innovative strategies for adapting PE to different diseases. This review critically examines the current evidence on the effects and feasibility of PE in GSDs and fatty acid oxidation disorders, focusing on the distinction between aerobic and anaerobic exercise. Additionally, it explores the usefulness of supporting nutritional strategies while identifying literature gaps. Finally, based on the available data, novel theories for exercise adaptation are discussed, aiming for prospective validation in future studies.

## Introduction

Metabolic myopathies are a diverse group of neuromuscular disorders that impair cellular energy metabolism, primarily affecting skeletal muscle (Berardo et al. 2010). These conditions result from genetic mutations that disrupt the production of adenosine triphosphate (ATP), a crucial energy molecule necessary for muscle contraction and function (Vissing 2016). Major energy sources for ATP production in muscle cells include muscle glycogen, blood glucose, and fatty acids derived from both intramuscular and adipose tissue (Hargreaves and Spriet 2020). In metabolic myopathies, defects in genes related to fatty acid oxidation, glycolysis, and glycogenolysis hinder the muscle’s ability to utilize the relative substrates, leading to insufficient ATP production to meet energy demands (Ørngreen and Vissing 2017; Olpin et al. 2015). Despite their varied origins and clinical presentations, many of these disorders exhibit common symptoms such as muscle weakness, early fatigue, myalgia, and rhabdomyolysis (RML). Importantly, RML is considered the most serious manifestation of these diseases due to its potential to release nephrotoxic substances into the bloodstream, which can cause acute kidney failure or even death (Rawson et al. 2017; Torres et al. 2015; McMahon et al. 2013). While factors such as heat, fasting, and infections act as predominant RML triggers mainly in fatty acid oxidation (FAO) disorders (Pennisi et al. 2018; Scalco et al. 2015), physical exercise (PE) is a common potential trigger of RML in both FAO disorders and Glycogen Storage Disorders (GSDs) (De Castro et al. 2015; Quinlivan et al., 2011; Lodin et al. 2024; Giannoglou et al. 2007). Consequently, except for diseases for which specific guidelines exist, like GSD type V (Lucia et al. 2021), clinical management often prioritizes PE prevention (Ørngreen and Vissing 2017; Olpin et al. 2015). However, while refraining from PE may help prevent RML and its complications in the short term, it can also lead to significant long-term negative consequences. Physical inactivity is associated with an increased risk of chronic conditions such as obesity, type 2 diabetes, cardiovascular diseases, sarcopenia, osteoporosis, and mood disorders (Booth et al. 2012; Dores et al. 2024; Bull et al. 2020; Warburton et al. 2006; Liao et al. 2015). Furthermore, prolonged inactivity may further worsen muscle function, impair quality of life (QoL), and negatively impact social life (Lodin et al. 2024; Senn et al. 2023). Therefore, identifying safe exercise modalities for these patients is critically important. Given the absence of disease-specific exercise guidelines for most of these conditions, reviewing existing literature can provide a foundation for a rationale worthy of being tested in future clinical trials to shed light on the possibilities of safely performing PE in the context of such disorders. This review aims to summarize and critically evaluate the available literature on the feasibility and effects of PE in patients with metabolic myopathies at risk of RML. Our objective is to suggest emerging theories regarding sustainable exercise modalities tailored to specific conditions, while drawing attention to existing gaps in the literature that warrant further exploration.

## Definition, etiology, and pathogenic mechanisms of rhabdomyolysis

**Fig. 1:**
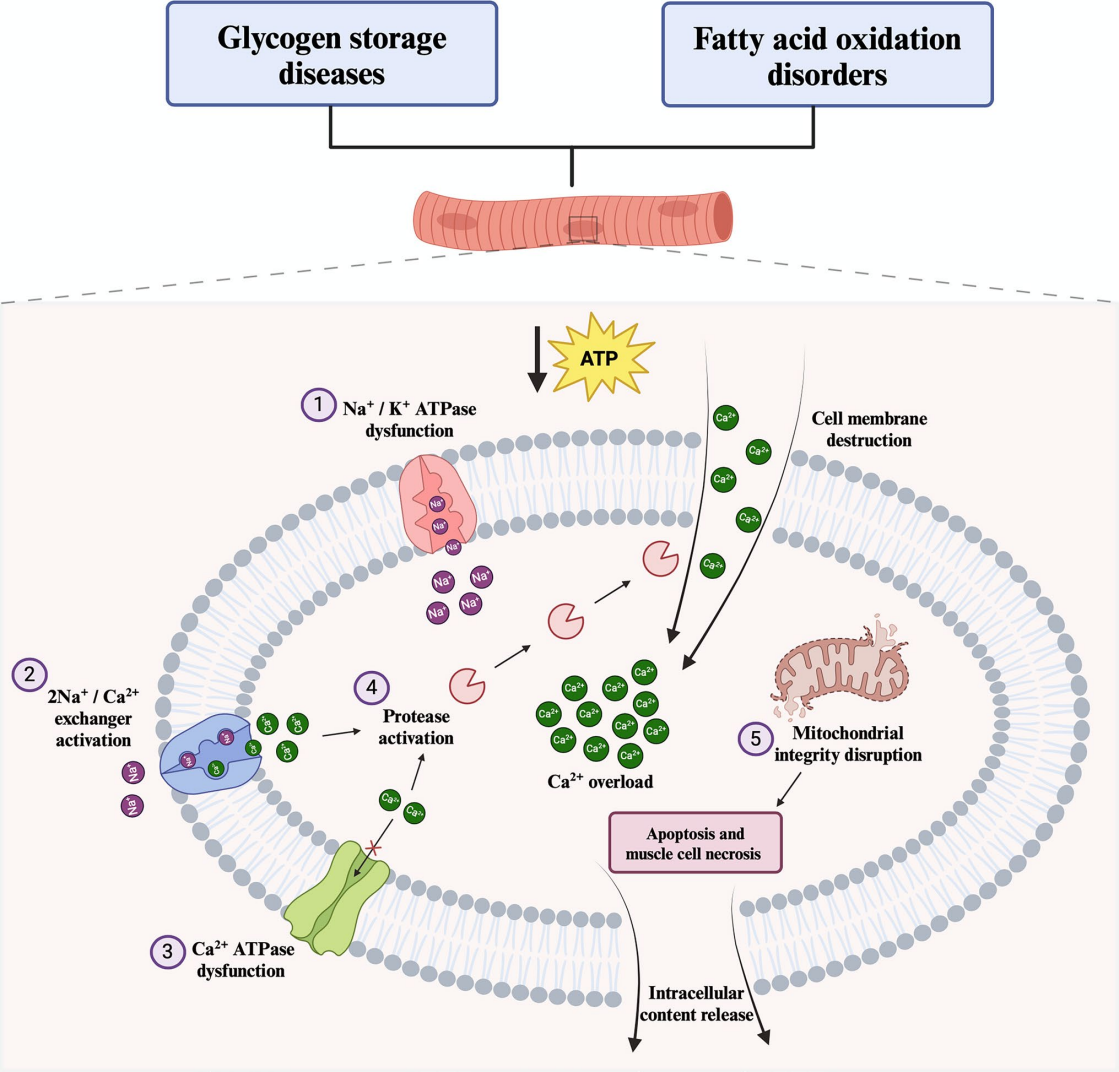
HYPERLINK "sps:id::fig1||locator::gr1||MediaObject::0" Possible mechanism underlying RML in metabolic myopathies. 1) The reduced availability of ATP would lead to dysfunction of the Na/K-ATPase pump, resulting in intracellular Na^+^ accumulation. 2) Intracellular Na^+^ accumulation activates the Na^+^/Ca^2+^ exchanger, which, in an attempt to compensate, expels Na^+^ and imports Ca^2+^. 3) The reduced availability of ATP would also cause dysfunction of the Ca^2+^-ATPase pump, impairing its ability to export Ca^2+^, leading to its intracellular accumulation. 4) Intracellular Ca^2+^ accumulation activates proteases that degrade the cell membrane, resulting in further Ca^2+^ influx. 5) Ca^2+^ overload disrupts mitochondrial integrity, which in turn induces apoptosis and cellular necrosis, leading to the release of intracellular content (e.g., CK) into the bloodstream. ATP, adenosine triphosphate; Ca^2+^, calcium; CK, creatine kinase; K^+^, potassium; Na^+^, sodium; RML, rhabdomyolysis.

RML, characterized by the destruction of skeletal muscle cells (Torres et al. 2015; Chavez et al. 2016), typically presents with muscle pain, weakness, myoglobinuria (often appearing as dark colored urine), and elevated serum creatine kinase (CK) levels, which is the most sensitive laboratory marker for its assessment (Huerta-Alardín et al. 2005). Although there is no consensus on a specific CK threshold indicative of RML, a level ten times higher than baseline is generally accepted as a diagnostic criterion (Lee 2014). Additionally, CK values exceeding five times the baseline or greater than 5000 U/L are also considered indicative of RML (Torres et al. 2015; Nance and Mammen 2015). Noteworthy, RML is inherently context dependent and requires a clearly defined clinical or physiological framework to be meaningful. In fact, while acute RML, which often involves myoglobinuria and systemic manifestations, represents a clinically significant risk, chronic CK elevations can be frequently observed in various metabolic myopathies (Rouyer et al. 2024) and are not usually accompanied by such systemic involvement. Acute kidney injury is the most common complication associated with RML and is typically treated with prompt fluid therapy (Chatzizisis et al. 2008). While this condition is generally reversible, severe cases can lead to complications with high mortality rates (Chatzizisis et al. 2008). As previously mentioned, in metabolic myopathies, RML can be triggered by various etiological factors; however, symptoms primarily arise during PE and are mainly due to metabolic stress (Ørngreen and Vissing 2017). This stress results from a mismatch between energy demand and supply, initiating a cascade of events that ultimately lead to muscle cell necrosis and the release of intracellular contents into the bloodstream (Ørngreen and Vissing 2017; Chavez et al. 2016). Regardless of the triggering factors, the pathogenic mechanism is characterized by an excessive increase in intracellular calcium (Ca^2^⁺) and sodium (Na⁺) concentrations, which might result from impaired ATP production (Torres et al. 2015; Chavez et al. 2016; Hamel et al. 2015). The homeostasis of Ca^2^⁺ and Na⁺ in muscle cells is maintained by energy-dependent pumps, such as the Na⁺/K⁺ ATPase pump (Pirkmajer and Chibalin 2016). This pump helps maintain cell volume and membrane potential by exporting three Na⁺ ions out of the cell and importing two K⁺ ions in an ATP-dependent manner (Pirkmajer and Chibalin 2016). A reduction in ATP availability could decrease pump activity (Chavez et al. 2016), leading to an accumulation of intracellular Na⁺, which draws water into the cell, disrupts intracellular integrity, and activates the Na⁺/Ca^2^⁺ exchanger. To restore electrolyte balance, this exchanger increases intracellular Ca^2^⁺ levels (Chavez et al. 2016). Due to ATP depletion, the ATP-dependent Ca^2^⁺ pump would fail to expel excess Ca^2^⁺ ions, resulting in sustained myofibrillar contraction, further ATP depletion, and the activation of proteases, such as phospholipase A2 (Torres et al. 2015; Chavez et al. 2016). This enzyme contributes to the destruction of the cell membrane, facilitating additional Ca^2^⁺ influx (Torres et al. 2015; Chavez et al. 2016). The resulting Ca^2^⁺ overload compromises mitochondrial integrity, inducing apoptosis (Chavez et al. 2016). These combined events lead to muscle cell necrosis and the subsequent release of cytotoxic components into the bloodstream (Torres et al. 2015; Chavez et al. 2016; Huerta-Alardín et al. 2005). It is important to note that, although the proposed ATP depletion model offers a coherent explanation for the intracellular events involved in RML (Torres et al. 2015; Chavez et al. 2016; Hamel et al. 2015), the assumption that it results solely from an energy deficiency remains speculative. Additional contributing factors and alternative mechanisms may also play significant roles in its development (for a review see de Calbiac et al. 2025). A summary of the theoretical pathogenic mechanisms of RML is provided in Fig. 1.

## Methodology

In this study, we focused on English-language articles that examine a subset of myopathies where PE is a known risk factor for RML, primarily following the classification proposed by Scalco et al. (2015). Metabolic myopathies are generally divided into two main categories: GSDs, which affect muscle carbohydrate metabolism, and FAO disorders, which impact muscle lipid metabolism. While specific PE guidelines have already been proposed for GSD V (Lucia et al. 2021), we have chosen to report the existing evidence to support the identification of a rationale for exercise adaptation which may also apply to other disorders with similar pathophysiological features. To gather relevant literature, we conducted searches in the PubMed and Web of Science databases using a combination of keywords and major subject headings. These included terms such as “physical exercise,” “training,” “myophosphorylase deficiency,” “McArdle disease,” “phosphofructokinase deficiency,” “Tarui disease,” “phosphorylase kinase deficiency,” “phosphoglycerate mutase deficiency,” “beta-enolase deficiency,” “phosphoglucomutase 1 deficiency,” “phosphoglycerate kinase-1 deficiency,” “carnitine palmitoyl-transferase II deficiency,” “long-chain 3-hydroxyacyl-CoA dehydrogenase deficiency,” “very-long-chain acyl-CoA dehydrogenase deficiency,” “glutaric aciduria type II,” “lipin-1 deficiency,” “creatine kinase,” and “rhabdomyolysis.” It is important to note that this review did not use a systematic search strategy; therefore, some relevant literature may have been overlooked. Only studies reporting exercise-induced variations in CK, as a key biomarker of RML risk (Huerta-Alardín et al. 2005), were eligible for inclusion. This criterion applied to both structured training protocols and isolated exercise sessions, encompassing group studies as well as case reports. Given the focus on exercise-induced RML, we concentrated on studies involving older children and adults, in whom this clinical presentation is more common, whereas neonatal and infantile forms usually present with hypotonia and severe multisystem involvement (Tobon 2013). In the first part of the present work, we report, where present, the types of exercise and nutritional protocols proposed across different diseases, along with the associated changes in CK levels. Following, in the Discussion section, we provide a critical evaluation of the available literature and propose theoretical emerging principles for exercise adaptation.

## Glycogen storage diseases

### Myophosphorylase deficiency (McArdle disease)

Myophosphorylase deficiency, also known as GSD V or McArdle disease, is a rare autosomal recessive disorder that affects muscle metabolism, occurring in approximately 1 in 100,000 to 167,000 individuals among Caucasians (Godfrey and Quinlivan 2016). It is caused by mutations in the *PYGM* gene on chromosome 11, which encodes the enzyme glycogen phosphorylase (myophosphorylase) in muscle tissue (De Castro et al. 2015; Santalla et al. 2017). This enzyme is crucial for breaking down glycogen into glucose-1-phosphate (Lucia et al. 2008). Due to impaired glycogen breakdown, patients with GSDV struggle to resynthesize ATP during PE, resulting in a mismatch between energy demand and substrate availability that leads to exercise intolerance (Dimauro et al. 2002). Muscle fatigue typically manifests within 3–5 min of aerobic exercise, peaking at around 6–8 min (Haller et al. 2006). However, current guidelines (Lucia et al. 2021) indicate that patients can sustain PE due to the “second wind” phenomenon, which enables a metabolic shift toward alternative energy substrates such as fats and liver-derived glucose (Haller and Vissing 2002). With appropriate nutritional strategies (pre-exercise glucose ingestion) and operational measures (such as a light warm-up, controlled intensity, and adequate rest), both aerobic and resistance training can be performed safely. Of note, glucose supplementation during exercise appears unnecessary, as suggested by the lack of additional benefit from repeated sucrose ingestion over placebo in terms of HR or perceived exertion after the second wind (Løkken et al. 2023). Specifically, it is reported that aerobic exercise should begin at very low intensity and be gradually increased following the onset of the second wind. For resistance training, brief maximal efforts (< 6 s), followed by full recovery (~ 3 min), take advantage of the preserved phosphagen system, thereby minimizing the risk of RML. In our review, we analyzed nine studies that assessed the effects of aerobic, anaerobic, or mixed training programs, both with and without accompanying nutritional interventions.

#### Aerobic training

In a study conducted by Haller et al. (2006), eight subjects (four males and four females, aged 33–61 years) were evaluated to determine the safety and feasibility of aerobic exercise in individuals with GSD V. The training program consisted of cycle ergometer exercises performed four times per week over a period of 14 weeks, with each session lasting 30 to 40 min at an intensity of 60–70% of the maximum heart rate (HR). To assess the effects of the training program, participants underwent cardiopulmonary exercise testing (CPET) before and after the intervention. The results indicated a significant improvement, with a 14% increase in maximal oxygen uptake (V̇O₂max) and a 15% rise in peak cardiac output following the training. Notably, throughout the program, patients reported no muscle pain or cramps. Additionally, CK levels, monitored weekly in some patients during the training protocol, remained stable 24 h post-exercise.

#### Aerobic training combined with a nutritional approach

In the study conducted by Maté-Muñoz et al. (2007), nine patients (four males and five females, aged 17 to 69 years) participated in an eight-month supervised aerobic training program composed by walking or cycling sessions 5 times per week at a low-to-moderate intensity, capped at 60% of their maximum HR. The duration of each session gradually increased from 10 to 60 min. In conjunction with the exercise regimen, participants were instructed to consume 100 g of complex carbohydrates one hour prior to each session and a 330 ml solution containing 30 g of simple carbohydrates during the warm-up. This combined approach of exercise and nutritional intervention led to significant improvements in several key metrics. Notably, there was an increase in peak oxygen uptake (V̇O₂peak) and ventilatory threshold, indicating enhanced aerobic capacity and endurance. Importantly, the study also reported a decrease in CK levels between pre- and post-training assessments. CK levels dropped significantly both at rest (from 2235.8 ± 2082.1 U/L to 595.1 ± 1074.3 U/L) and after exercise (from 2283.8 ± 1875.6 U/L to 711.4 ± 1104 U/L).

Similarly, in the study by Lucia et al. (2007), a 29-year-old female patient with GSD V participated in a three-month aerobic training program that included five low-to-moderate intensity (60% of her maximum HR) exercise sessions per week. The adopted exercise modality was walking. The duration of the sessions gradually increased, reaching 60 min. Like the protocol used in the study by Maté-Muñoz et al. (2007), participant was advised to consume 100 g of complex carbohydrates one hour prior to exercise and 30 g of simple carbohydrates during the warm-up. The results of this training program were promising, with the patient demonstrating a remarkable 44% improvement in exercise time and a 50% increase in V̇O₂peak. Additionally, CK levels were assessed both before and after exercise, showing initial values of 5278 U/L and 5443 U/L, respectively. Following the training regimen, CK levels decreased by an impressive 92%, indicating significant improvements in muscle endurance and a reduced risk of muscle damage.

In the study conducted by Pérez et al. (2007), a 38-year-old male patient with GSD V participated in a seven-month supervised aerobic training program that included 3–4 running sessions per week at an intensity less than or equal to 80%–85% of maximum HR. The training regimen began with 10 min of running, gradually increasing to a duration of 60 min. Consistent with previous studies, the nutritional approach involved consuming 100 g of complex carbohydrates one hour prior to exercise and 30 g of simple carbohydrates during the warm-up. Throughout the training program, CK levels were monitored, revealing a significant reduction. Baseline CK levels were recorded at 5,682 U/L and post-exercise levels at 5,700 U/L before the intervention. After completing the training program, baseline CK levels decreased to 2,641 U/L, with post-exercise levels at 2,805 U/L. Additionally, the patient experienced substantial improvements in physical performance metrics: V̇O₂peak increased from 14.6 to 30.8 ml/kg/min, indicating enhanced aerobic capacity. Furthermore, gross muscle efficiency, defined as the ratio between external mechanical work produced and total metabolic energy consumed during activity, improved from 13.8% to 17.2%.

In the study by Pérez et al. (2008), the clinical progression of an 8-year-old male patient with GSD V was monitored over the course of one year. Following a preliminary maximal exercise treadmill test, the patient was advised to engage in moderate physical education, which included 2–3 sessions per week of non-competitive swimming and 1–2 classes per week of physical education, while avoiding exercises that involved high muscle loads. To support his energy needs and prevent drops in blood glucose levels throughout the day, the patient was recommended to consume 100 g of complex carbohydrates during breakfast and lunch. Additionally, he was instructed to take a 250 ml solution containing 20 g of simple carbohydrates during the warm-up period before his physical education and swimming classes. After one year of adhering to this combined approach of moderate exercise and nutritional support, the patient's baseline CK levels showed a significant decrease from 1570 U/L to below 251 U/L.

#### Anaerobic training

In the study conducted by Pietrusz et al. (2018), the effect of a resistance training program was evaluated in two male patients with GSD V, aged 37 and 46, which will be referred to as patient 1 and patient 2, respectively. Patient 1 followed a four sessions week program over a period of 4 years, composed by exercises mainly performed using free weights compound movements, with sets of 1–5 repetitions and a variable resting time of 2–5 min between sets (depending on the percentage of 1RM). Occasionally, he tried to perform sets of 4 repetitions with a rest of 30 s. Patient 2 followed a protocol based on the use of resistance machines over a period of 3 months, with sets of 5 to 15 repetitions per exercise, stopping as soon as muscle discomfort occurred, and 1 min rest between sets. Throughout the duration of the training periods, neither patient experienced myoglobinuria, nor did they show significant increases in CK levels. Notably, patient 1 showed a remarkable decrease in CK levels over the course of the training period. Specifically, his mean CK levels dropped from 3006 U/L during the years 2011–2014 to 1029 U/L from 2015–2017, with a further reduction to 941 U/L assessed in July 2017. The authors of this work suggest that short bouts of resistance exercises lasting no longer than 10 s preceded and followed by rest periods of between 30 s and 3 min may be safely performed by MD patients.

#### Anaerobic training combined with a nutritional approach

In the study by García-Benítez et al. (2013), a combined resistance training and nutritional protocol was implemented for a 15-year-old male patient with GSD V over a period of six weeks. The training program consisted of two 60 min sessions per week, incorporating 2–3 sets of eight main exercises, with each set consisting of 10–15 repetitions at an intensity of 60–75% of the patient's one-repetition maximum (1 RM). The accompanying nutritional strategy aimed to optimize energy availability and performance. It included a diet that provided 65% of total energy from carbohydrates during breakfast and lunch prior to training sessions. Additionally, the patient consumed a 500 ml solution containing 40 g of simple carbohydrates five minutes before each training session. This combined approach resulted in significant improvements in strength performance: the patient experienced a 27% increase in one-repetition maximum bench press performance and a 6% improvement in multipower squat performance. Importantly, these gains were achieved without any episodes of myoglobinuria or significant increases in CK levels. Furthermore, CK levels measured 24 h after the one-repetition maximum test showed a notable decrease from 3636 U/L pre-training to 1575 U/L post-training.

One year later, in a study conducted by Santalla et al. (2014), seven patients with GSD V (five women, mean age of 38.4 years) participated in a 16-week anaerobic circuit training program. The training sessions were held twice a week and involved performing 4 exercises, each consisting of 4–6 or 6–8 sets with 5–6 repetitions per set, at a perceived exertion level of 6–7, which indicates a moderate to high intensity. Alongside the exercise regimen, participants followed a nutritional protocol similar to that used in the previous study by García-Benítez et al. (2013). This included consuming 100 g of complex carbohydrates at breakfast and lunch prior to training sessions, and a 330 ml solution containing 30 g of simple carbohydrates pre-exercise. The results of the training program indicated an increase in total and lower body lean mass, as assessed by dual energy X-ray absorptiometry (DEXA). Interestingly, after a two-month detraining period following the completion of the program, muscle mass reverted to pre-training levels. Additionally, CK levels remained unchanged throughout the study.

#### Mixed training combined with a nutritional approach

In the study conducted by Santalla et al. (2022), the effects of a two-year exercise program were evaluated in ten patients with GSD V (six males and four females, with a mean age of 38 years). The exercise regimen consisted of five sessions per week of moderate aerobic training, each lasting one hour, performed on a stationary cycle at an intensity corresponding to a Borg rating of perceived exertion (RPE) scale of 5–7. In addition to aerobic training, participants engaged in strength training sessions two to three times per week, ensuring a minimum recovery period of 48 h between consecutive strength training sessions. Each anaerobic training session consisted of 3 circuit laps. Each lap included 3 sets of 4 exercises, with 6 repetitions per set. The intensity was maintained at 6–7 on the OMNI-RPE scale (Robertson et al. 2003) and 0–1 on the RPP scale (Jensen and McFarland 1993). Rest intervals of 2–3 min were allowed between sets. To support their training, patients were advised to consume 100 g of complex carbohydrates one hour before each session. Additionally, they ingested a 250 ml solution containing 20 g of simple carbohydrates at the end of the warm-up phase. The results indicated that there were no significant increases in CK levels or episodes of myoglobinuria during or after the training sessions. Importantly, the exercise program led to notable functional improvements compared to a control group that engaged in low-intensity walking exercises for either 30 min or one hour without any resistance training throughout the study period. The improvements observed in the exercise group included increased ventilatory threshold, V̇O₂peak, and peak workload.

### Phosphofructokinase deficiency (Tarui disease)

Muscle phosphofructokinase (PFK) deficiency, also known as Tarui disease or GSD VII, is a rare autosomal recessive disorder that significantly impacts glycogen metabolism. This condition typically manifests in childhood and is characterized by a range of symptoms, including exertional fatigue, severe muscle exercise intolerance, compensated hemolysis, hyperuricemia, and occasionally myoglobinuria, which can increase the risk of acute kidney failure. To date, approximately 100 cases have been documented globally, with a particularly severe infantile form reported in six families (Vives-Corrons et al. 2013). The underlying cause of PFK deficiency is mutations in the PFKM gene located on chromosome 12q13. This gene encodes the muscle-specific isoenzyme of PFK, an essential enzyme that regulates anaerobic glycolysis. Muscle biopsy of affected individuals reveals abnormal glycogen accumulation and reduced PFK activity, ranging from 1 to 33%. In contrast, erythrocyte PFK activity remains above 50%, which helps differentiate this condition from other metabolic disorders. Patients with PFK deficiency are considered to be at high risk for exercise-induced RML, and baseline CK levels are often elevated in these individuals (Scalco et al. 2015). Of note, although the guidelines on GSD V also mention this condition (Lucia et al. 2021), the authors report that the available data are insufficient to provide definitive indications regarding PE feasibility. It is noteworthy that there have been no published intervention studies specifically assessing CK levels following training programs or exercise sessions in this population. Two studies have investigated affected individuals using a cycle ergometer to evaluate their exercise capacity and response to physical activity (Haller and Lewis 1991; Vissing et al. 1996). Haller and Lewis (1991), analyzing four patients (three males aged 18, 22, and 48 years old, and one female aged 17 years old), reported that glucose infusion reduces the oxidative capacity of muscle and the capacity for aerobic exercise, while Vissing et al. (1996), analyzing two males and one female patients (aged 55, 24 and 23 years old, respectively), described a paradoxically exaggerated mobilization of glucose during exercise. However, as they did not report CK levels or other markers indicative of muscle damage, these studies did not meet the inclusion criteria for the present review.

### Phosphorylase kinase deficiency

Muscle phosphorylase kinase (PhK) deficiency, also known as GSD IXd, is a rare genetic disorder affecting glycogen metabolism, with fewer than 30 cases documented in the literature (https://www.orpha.net/en/disease/detail/715?name=gsd%209&mode=name. Accessed March 5, 2025). This condition arises from mutations in the *PHKA1* gene, which encodes the muscle-specific isoform of the alpha subunit of PhK. Of note, PhK is a critical enzyme involved in glycogenolysis, the process by which glycogen is broken down into glucose for energy. The PhK enzyme complex is multimeric and consists of four subunits: alpha, beta, gamma, and calmodulin. Each of these subunits is encoded by different genes and expressed in various tissues, contributing to the complexity of its regulation and function (Geramizadeh et al. 2024). GSD IXd typically presents during adolescence or adulthood with symptoms such as exercise intolerance, myalgia, cramps, fatigue, and myoglobinuria. In some patients, progressive muscle weakness may develop over time, while others may experience mild symptoms or remain asymptomatic. A rare neonatal form has also been described, characterized by generalized muscular hypotonia and respiratory insufficiency. Despite the generally mild nature of symptoms in many individuals with GSD IXd, this condition is considered to carry a risk for exercise-induced RML, which can lead to serious complications (Scalco et al. 2015). Notably, there have been no published intervention studies that assess the effects of training programs on this patient population. The existing literature includes two studies that investigated exercise sessions among a total of 3 individuals (one 50-year-old female and two males aged 39 and 69 years, respectively) with GSD IXd (Ørngreen et al. 2008; Preisler et al. 2012), both suggesting a normal lactate increase during anaerobic forearm exercise, a blunted or absent lactate response during aerobic cycle submaximal exercise, and an absence of the second wind phenomenon. However, these studies did not measure serum CK levels before and after exercise.

### Phosphoglycerate mutase deficiency

Phosphoglycerate mutase deficiency, also known as GSD X, is a rare metabolic myopathy characterized by fewer than 50 reported cases (https://www.orpha.net/en/disease/detail/97234?name=gsd%2010&mode=name). Accessed March 5, 2025. This condition arises from a deficiency in phosphoglycerate mutase (PGAM2), an enzyme crucial for glycolysis, which is the metabolic pathway that converts glucose into pyruvate to produce ATP, the energy currency of the cell. Patients with GSD X typically experience exercise-induced RML, muscle pain, cramps, and myoglobinuria. The underlying mechanism involves mutations in the *PGAM2* gene that lead to reduced enzyme activity during physical exertion. This impairment disrupts glycolysis and ATP production, ultimately resulting in muscle breakdown and associated symptoms. Clinical features of GSD X include elevated CK levels, glycogen accumulation in muscle tissue, and the presence of tubular aggregates observed on muscle biopsy. Additionally, patients often exhibit a reduced lactate response during ischemic exercise tests, indicating impaired anaerobic metabolism (Oh et al. 2006). While there have been case reports detailing the symptoms and clinical histories of individuals with GSD X, no specific training protocols tailored for this population have been established. A study conducted by Vissing et al. (2005) involved two unrelated male patients aged 21 and 26 who performed cycling at 65% of their V̇O₂max, followed by incremental exercise until exhaustion. The study show that, despite lactate, glucose, and intralipid infusions administered on separate days during the exercise protocols, neither patient experienced a second wind phenomenon or improvement in exercise capacity, highlighting substantial differences compared to patients with McArdle disease. Notably, CK levels were not assessed during this study.

#### Exercise sessions

The only study to assess the effect of PE in a 24-year-old male patient with this disease was conducted by Kissel et al. (1985). The study involved two incremental treadmill tests lasting 18 min, during which speed and inclination were increased every minute, starting from 1.5 mph up to 5.5 mph and from a 4% inclination to 20%. CK levels were measured before the test and two minutes after its completion, reporting a negligible increase in CK on both occasions (from 64 to 70, from 50 to 55 U/mL, respectively).

### Beta-enolase deficiency

Beta-enolase deficiency (GSD XIII) is a rare inherited metabolic myopathy caused by autosomal recessive mutations in the *ENO3* gene, which encodes the beta-enolase enzyme, a critical component of glycolysis in skeletal muscle. Beta-enolase catalyzes the conversion of 2-phosphoglycerate to phosphoenolpyruvate, and its deficiency leads to impaired energy production in muscle cells (Comi et al. 2001). Clinically affected individuals present exercise intolerance, episodic RML, and muscle pain (Tarnopolsky 2018). The first patient with a benign phenotype characterized by exercise intolerance was presented by Comi et al. (2001). This patient experienced myalgia after physical exertion, mild hyperCKemia, but no urine discoloration or pigmenturia. Beyond this specific case, only five cases of GSD XIII have been reported worldwide (Buch et al. 2021; Musumeci et al. 2014). There are no studies available for this disease that have considered a long-term training program.

#### Exercise sessions

A study by Buch et al. (2021) investigated exercise capacity and muscle metabolism in patients with GSD XIII, focusing on the metabolic response during PE and the effects of intravenous glucose infusion. The study included three men (aged 21, 41, and 50) with GSD XIII and 10 healthy controls. Participants underwent a handgrip strength test and a maximal cycle ergometer test to measure V̇O₂max. The following day, a one-hour submaximal exercise test was performed at 65%–75% of their V̇O₂max. Two days later, the submaximal exercise test was repeated with a 10% intravenous glucose infusion. Results showed that patients had lower V̇O₂max compared to controls, with two out of three patients prematurely discontinuing the maximal test. The glucose infusion did not enhance exercise capacity. Compared to rest values, CK levels at peak-maximal cycle ergometer test increased, but remained below the threshold for RML (patient 1: from 126 to 374; patient 2: from 931 to 2530; patient 3: from 94 to 121, respectively). No CK levels were reported before and after the submaximal exercise and handgrip tests. In conclusion, the patients included in the present study only showed a tendency toward increased CK levels, without approaching thresholds possibly indicative of RML. Moreover, they retained near-normal anaerobic metabolic responses during submaximal exercise. Based on these observations, the authors suggest that GSD XIII may represent one of the mildest metabolic myopathies affecting glycolysis.

### Phosphoglucomutase 1 deficiency

Phosphoglucomutase 1 (PGM1) deficiency, or GSD XIV, is a rare genetic disorder which can be classified as a congenital disorder of glycosylation and a glycogen storage disease, with a prevalence of less than 1 in 1,000,000 individuals (https://www.orpha.net/en/disease/detail/319646). Accessed March 5, 2025. PGM1 affects N-linked glycosylation, glycolysis, and glycogen storage. The PGM1 enzyme, located in the cytosol, is responsible for interconverting glucose-1-phosphate into glucose-6-phosphate (Radenkovic et al. 2024). Individuals with PGM1 deficiency can present with a broad spectrum of clinical manifestations, ranging from isolated myopathy to a multisystemic disorder with bifid uvula, malignant hyperthermia, hypogonadotropic hypogonadism, growth retardation, liver involvement, hypoglycemia, myopathy, dilated cardiomyopathy, endocrine and hematological abnormalities, and cardiac arrest (Radenkovic et al. 2024; Tegtmeyer et al. 2014; Altassan et al. 2021). Interestingly, similarly to GSD V, it has been reported that these patients may experience the second wind phenomenon (Preisler et al. 2017), which could potentially make PE feasible under specific conditions. Upon reviewing the literature, we identified three case reports assessing PE in this cohort of patients (Preisler et al. 2013, 2017; Voermans et al. 2017). However, only one met our inclusion criteria, as it specifically assessed exercise testing and muscle damage evaluated through CK levels.

#### Exercise sessions combined with a nutritional approach

In the study by Voermans et al. (2017), the effect of galactose supplementation, a recommended dietary treatment for patients with PGM1 deficiency (Altassan et al. 2021), on exercise capacity was evaluated in a 53-year-old male. The supplementation regimen consisted of 50 g of oral galactose per day, divided into four doses, over a six-month period. The patient underwent an incremental cycling test before and after the intervention. In each evaluation, CK levels were measured before the exercise test and the following day. Before treatment, CK levels increased from a baseline of 2,500 U/L to a peak of 13,600 U/L. After the intervention, baseline CK levels decreased to 875 U/L, and the post-exercise peak was reduced to 3130 U/L, indicating a significant reduction compared to pre-treatment values. Both peak and baseline CK levels showed substantial improvement following treatment. Notably, a similar reduction in CK levels was observed when comparing the response to a constant 30W submaximal workload cycle ergometry exercise before and after treatment. Specifically, CK levels decreased from 15,300 U/L to 3070 U/L.

### Phosphoglycerate kinase-1 deficiency

Phosphoglycerate kinase-1 (PGK-1) deficiency is a rare X-linked disorder characterized by a highly variable clinical phenotype that can include hemolytic anemia, myopathy, and neurological manifestations like intellectual disabilities (Turner et al. 1995; Fermo et al. 2012), epilepsy (Sugie et al. 1989), adult-onset parkinsonism (Sotiriou et al. 2010) and leukodystrophy (Baba et al. 2018). Affected individuals may exhibit one or more of these manifestations, highlighting significant heterogeneity in clinical presentation (Shirakawa et al. 2006). The myopathic condition is caused by mutations in the *PGK1* gene (Xq13.3), which encodes phosphoglycerate kinase (PGK), a key enzyme in the glycolytic pathway. PGK catalyzes the conversion of 1,3-bisphosphoglycerate into 3-phosphoglycerate, generating ATP. To date, approximately 20 pathogenic variants in the PGK-1 gene have been identified in about 30 unrelated affected families, making its overall prevalence unknown but likely very low (Prabhu et al. 2023). There is a tendency toward exertional myoglobinuria and muscle cramps, as well as recurrent RML, in relation to this disease (Scalco et al. 2015; Rosa et al. 1982; Hamano et al. 2000). To date, no studies have investigated the effects of training in this patient cohort.

#### Exercise sessions

The only study that tested the metabolic response to PE was conducted by Hogrel et al. (2019). The authors used the forearm non-ischemic exercise test in a 69-year-old man diagnosed with PGK-1 deficiency. To study the muscle behavior during 70% of the maximal grip strength sustained for 30 s, they observed levels of lactate, ammonia, and CK. The results showed a significant increase in ammonia levels, whereas lactate levels remained normal. After 24 h, the CK level increased from 544 U/L to 1,920 U/L, suggesting that this subject experienced muscle damage after a short-duration isometric exercise.

## Fatty acid oxidation disorders

### Carnitine palmitoyl-transferase II deficiency

Carnitine Palmitoyl-Transferase II (CPT II) deficiency is a rare inherited metabolic disorder that affects the mitochondrial oxidation of long-chain fatty acids (LCFA) (Negro et al. 2021). Three forms of CPT II deficiency have been described: a myopathic form, a severe infantile form, and a neonatal form. To date, over 300 cases of CPT II deficiency have been reported, with the myopathic form being the most common (myopathic form: 86%, severe infantile form: 8%, neonatal form: 6% of cases) (Wieser et al. 2003). The myopathic form is the least severe and is characterized by recurrent attacks of RML, muscle pain, and weakness triggered by prolonged PE, fasting, viral illness, or extremes in temperature (Isar et al. 2024). To date, about 100 CPT2 mutations have been discovered (Yao et al. 2023). In these patients, the residual CPT II activity is generally sufficient to sustain FAO at rest but inadequate to meet the metabolic demands of exercise (Ørngreen et al. 2005).

#### Anaerobic training combined with a nutritional approach

A case report by our research group (Parimbelli et al. 2021) explored the effects of a high-carbohydrate, low-fat diet combined with interval and resistance training on a 14-year-old girl with CPTII deficiency, marking the first investigation of training in a CPTII patient. Over a year-long study, the patient underwent three one-hour training sessions per week. The interval training was performed on a treadmill, alternating 1 min run at 70% of maximal HR and 5 min’ walk, with an incremental duration which started from 15 min (at the beginning of the training program) and was gradually increased up to 30 min (toward the end of the training program). Resistance training included 6 exercises with 3 sets, each composed by 8 repetitions at 6–7 of the OMNI scale, with 2 min rest between sets. The nutritional intervention included 65% of medium and low glycemic index carbohydrates, 20% of fats (as extra virgin olive oil and medium-chain triglycerides (MCT) and 15% of high-biological-value proteins. Additionally, before, during, and after training sessions the patient was instructed to consume 500 ml of a solution containing 22.4 g of carbohydrates, 3 g of creatine, 248 mg of sodium, and 146 mg of potassium. After six months, the participants experienced slight weight and body mass index (BMI) gains, a decrease in the respiratory exchange ratio (RER) at rest and increases in peak power output, aerobic capacity, anaerobic threshold, and oxygen pulse during exercise. Levels of CK measured before and 36 h after two randomly selected sessions showed no significant changes, indicating no muscle damage (pre-313 U/L, post-314 U/L; pre-439 U/L, post-242 U/L, respectively). The study suggested that a high-intensity, short-duration training program was safe and beneficial for improving metabolic function and aerobic fitness in a CPT II deficiency patient.

Such a result seems in line with a subsequent study (Crisafulli et al. 2024a) in which we described the case of an 18-year-old male competitive basketball player affected by the disease. Based on an analysis of the macronutrients consumed during an incremental CPET, recommendations were provided to the patient regarding the exercise intensity (quantified both as % V̇O₂max and HR) to maintain in order to bypass fat oxidation as an energy substrate. Specifically, the analysis revealed that at 37.23% of V̇O₂max, corresponding to an HR of 111 bpm, the patient's fat oxidation was zero. Therefore, he was instructed to maintain this HR or higher during active exercise phases. The patient was also recommended to follow a diet with 50–55% of carbohydrates, 20% of fats (of which 60–75 ml/day of MCTs), and 20–25% of proteins, along with a supplementation with 3 g/day of *β*-alanine, 3 g/day of creatine, and with a carbohydrate’s solution (with 2:1 glucose:fructose ratio) during training/game sessions. The patient was then monitored for 5 months with periodical CK checks, showing that he no longer experienced RML while continuing competitive sports activity. Based on this observation, it seems possible that basketball, an activity characterized by short-duration, high-intensity bursts of exercise, if accompanied by proper nutritional strategy, could be a feasible form of PE for these patients, as it allows them to avoid relying on fat for energy production, an observation coherent with that reported by Parimbelli et al. (2021).

### Long-chain 3-hydroxyacyl-CoA dehydrogenase deficiency

Long-chain 3-hydroxyacyl-CoA dehydrogenase (LCHAD) deficiency is a mitochondrial disorder of fatty acid metabolism, characterized by hypoketotic hypoglycemia, hepatopathy, RML, cardiomyopathy, and an increased risk of coma, often presenting early in infancy (Spiekerkoetter et al. 2009). It results from mutations in the *HADHA* gene, which encodes the alpha subunit of the mitochondrial trifunctional protein (TFP), with the c.1528G > C mutation being the most common among European patients. The global prevalence is approximately 1 in 250,000 live births, but higher rates are found in regions around the Baltic Sea (https://www.orpha.net/en/disease/detail/5?name=Long-chain%203-hydroxyacyl-CoA%20dehydrogenase%20deficiency&mode=name). Accessed March 5, 2025. Management of LCHAD deficiency is challenging, especially during PE or prolonged fasting; however, MCT supplementation can help mitigate symptoms (Karall et al. 2014; van Eerd et al. 2017). There are currently no interventional studies on training protocols specifically for LCHAD. Nonetheless, a few studies have investigated the effects of a single session of aerobic exercise, often combined with nutrient supplementation to compensate for impaired energy substrate utilization. One study suggested that pre-exercise MCT supplementation improves exercise tolerance compared to carbohydrates but, CK evaluation was not performed (Behrend et al. 2012). After reviewing the literature, we identified two case reports that implemented few exercise sessions in patients with LCHAD and evaluated CK as an indicator of muscle damage.

#### Exercise sessions combined with a nutritional approach

Karall et al. (2014) conducted an endurance exercise test as part of a case report involving a 13-year-old boy with LCHAD deficiency. The patient followed a diet that included heptanoate supplementation (0.6 g/kg/day, accounting for 15% of total energy intake) and consumed a high-energy drink (containing hydrolyzed protein and fiber, providing 300 kcal per serving) before exercise. The study involved an incremental exercise test and three endurance exercise sessions lasting 30 min each at intensities of 80, 90, and 100 Watts. Throughout the protocols, the researchers monitored glucose, lactate, and acylcarnitine levels. Remarkably, clinical and biochemical parameters remained stable during the tests, with no signs of RML or increases in CK, indicating that the dietary interventions may have effectively supported the patient's metabolic needs during exercise. Similar findings were reported by van Eerd et al. (2017) in a 34-year-old female patient. The subject underwent three incremental CPET (comprising 4 min of unloaded cycling warm-up followed by an increasing workload of 1 Watt every 5 s at a pedal frequency of 60–80 RPM until exhaustion) over the course of one month to evaluate the effects of MCT supplementation on exercise tolerance and cardiac function, aiming to establish a safe training regimen with appropriate dietary recommendations. Blood samples were collected at rest, immediately before reaching the ventilatory threshold, and at maximal power output. The first test was conducted without MCT supplementation, the second with 10 g of MCTs, and the third with 20 g of MCTs. The supplement was administered 25 min prior to the test, while the patient’s habitual diet remained unchanged, and body weight remained stable throughout the testing period. Beyond a marked improvement in exercise performance, MCT supplementation resulted in a significant reduction in CK levels, both at rest (183, 135, and 110 iU/L, respectively) and during exercise (197, 139, and 115 iU/L, respectively). This observation further supports the potential role of MCT supplementation in facilitating exercise tolerance in patients with LCHAD deficiency.

### Very-long-chain acyl-CoA dehydrogenase deficiency

Very-long-chain acyl-CoA dehydrogenase (VLCAD) deficiency is a rare metabolic disorder caused by mutations in the *ACADVL* gene, which impair the mitochondrial beta-oxidation of long-chain fatty acids (Liang and Nishino 2010). The disease has an incidence of 1 in 31,500 (Arnold et al. 2009) and presents in three major phenotypes: severe infantile, intermediate childhood, and adult-onset myopathic forms. The severe infantile form, which presents within the first year of life, is associated with hypoketotic hypoglycemia, cardiomyopathy, liver dysfunction, and high mortality, often due to cardiac complications (Liang and Nishino 2010). The intermediate childhood form involves similar metabolic disturbances but with lower mortality. The adult-onset muscular form is characterized by recurring bouts of RML generated by prolonged activity or fasting (Fatehi et al. 2020). Research by Ørngreen et al. (2004) showed that VLCAD deficiency patients cannot increase FAO during exercise, relying instead on muscle glycogenolysis to meet energy demands. This reliance, alongside fatty acid intermediate accumulation, may lead to exercise-induced symptoms. Fatehi et al. (2020) reported six cases of VLCAD deficiency, with three showing exercise intolerance, four exhibiting RML and myoglobinuria, one with isolated hyperCKemia, and one with fatigue and weakness but normal CK levels. Patients are advised to avoid prolonged exercise and fasting (Herrera-Olivares et al. 2019). Since the first myopathic case was identified in 1994 (Ogilvie et al. 1994), three studies have investigated PE in VLCAD deficiency patients: one included anaerobic training (Herrera-Olivares et al. 2019), while two focused on aerobic tests (Diekman et al. 2016; Bleeker et al. 2020), each assessing muscle damage through specific biomarkers.

#### Anaerobic training combined with a nutritional approach

In a study by Herrera-Olivares et al. (2019), a 23-year-old female patient with a history of recurrent RML underwent a 6-month training program consisting of one month of high-intensity interval training (HIIT) (2 sessions per week) followed by 5 months of combined training (4 sessions per week, alternating 2 days of HIIT and 2 days of resistance training). HIIT sessions included 6 high-intensity sets (each of 70–80 s at a pedal frequency of minimum100 rpm) performed on a cycle ergometer, with 1 min rest between sets. The resistance training sessions consisted of 6 exercises performed as 2 circuit laps of 4–7 repetitions, at an intensity of 5–7 on the OMNI scale, including 2 min of rest between sets and 4 min of rest between laps. One hour before the session, the patient consumed 500 ml of a solution containing 30 g of carbohydrates and 15 g of MCTs. The results showed a significant increase in V̇O₂peak (+ 90.2%), maximal power output (+ 71.4%), and maximal HE (+ 20.6%), with no episodes of RML, muscle pain, or contractures. The study suggests that an anaerobic training program may be safely implemented in patients with VLCAD deficiency.

#### Exercise sessions

Diekman et al. (2016) investigated muscle ATP homeostasis in five VLCAD deficiency patients (four males and one female, age range 13–37 years) and 5 healthy controls during a 45-min cycling test at FATMAX intensity, which is the exercise intensity at which maximal FAO occurs (Venables et al. 2005), using metabolic monitoring and phosphorus magnetic resonance spectroscopy on the vastus lateralis muscle of the right leg. During exercise at FATMAX intensity, HCs exhibited only minor changes in phosphocreatine (PCr) and inorganic phosphate (Pi) concentrations (ΔPCr: –2.0 ± 0.7 mM; ΔPi: + 2.6 ± 1.0 mM) and maintained intramuscular pH above 7.0. In contrast, patients showed markedly greater PCr depletion and Pi accumulation (ΔPCr: –7.9 ± 1.0 mM; ΔPi: + 8.8 ± 0.8 mM). Changes in intramuscular pH among patients were more variable: while one patient maintained pH above 7.0, others showed progressive acidification during exercise, with pH values dropping to as low as 6.7 in some cases. While mitochondrial ATP synthesis remained intact, altered muscle fiber recruitment likely contributed to their reduced energy capacity. CK levels increased in two out of five patients: one patient exhibited a rise from 156 U/L to 576 U/L, while another showed an increase from 36 U/L to 400 U/L, an elevation that, according to the criteria proposed by Lee et al. (2014), would be indicative of RML. Anyhow, it must be considered that, in absence of myoglobinuria, it can be contextualized in the frame of the changes due to physiological workload (Radišić Biljak et al. 2025).

#### Exercise sessions combined with a nutritional approach

Bleeker et al. (2020) conducted a randomized, double-blind study involving five VLCAD deficiency patients (four males, age range 17–45 years). After a preliminary graded CPET to establish individual FATMAX, patients were randomly assigned to perform two exercise sessions, separated by at least one week. In each session, after consuming either a drink containing ketone esters and dextrose (KE + CHO) or an isocaloric drink containing only dextrose (CHO), patients performed 35 min on a cycle ergometer at their individual FATMAX, followed by 10 min of supine cycling in a magnetic resonance (MR) scanner. At FATMAX intensity (~ 40% V̇O₂max), MR spectroscopy revealed a 40% lower Pi/PCr ratio in the KE + CHO group, suggesting improved energy efficiency during exercise. Notably, post-exercise CK levels did not significantly increase in any subject, indicating that the same exercise modality proposed by Diekman et al. (2016) could be sustained through pre-exercise ingestion of ketone esters and dextrose in these patients. This suggests that nutritional strategies may help mitigate the risk of muscle damage during exercise in individuals with VLCAD deficiency.

### Glutaric aciduria type II

Glutaric aciduria type II (GA II), with a prevalence of 1–9 in 1,000,000 individuals (https://www.orpha.net/en/disease/detail/26791?name=ETFA&mode=gene). Accessed March 5, 2025, is a recessively inherited disorder affecting fatty acid and amino acid metabolism. It is caused by defects in two mitochondrial enzymes, namely electron transfer flavoprotein (ETF) and ETF-ubiquinone oxidoreductase (ET-QO), which are involved in the transfer of electrons (Frerman and Goodman 1985). Due to the inability to oxidize fatty acids and proteins, the disease typically leads to increased production of organic acids, resulting in metabolic acidosis (Scalco et al. 2015). Clinically, GA II manifests in three main phenotypes: neonatal-onset forms, which present with hypotonia, hepatomegaly, hypoglycemia, and high mortality with or without congenital abnormalities; and late-onset forms that exhibit a broad spectrum of symptoms, including myopathy, muscle weakness, and exercise intolerance (Scalco et al. 2015). While GA II patients are at risk for exercise-induced RML (Scalco et al. 2015), to the best of our knowledge, no studies have examined the effects of training or exercise sessions in this cohort of patients.

### Lipin-1 deficiency

Mutations in the lipin-1 gene (LPIN1), inherited in an autosomal recessive manner, are recognized as the most common cause of RML in pediatric patients (Michot et al. 2010). These mutations lead to the loss of lipin proteins, which are expressed at varying levels across different tissues, with lipin-1 being particularly prevalent in skeletal muscle, liver, and adipose tissue (Donkor et al. 2007). Lipin-1 plays a dual role as phosphatidate phosphatases, essential for triglyceride and phospholipid biosynthesis, and as transcriptional co-regulators involved in lipid metabolism and mitochondrial function (Csaki and Reue 2010; Zhang et al. 2014). Reduced lipin-1 levels impair fatty acid oxidation and energy production, particularly in skeletal muscle, leading to metabolic inflexibility and increased susceptibility to RML under metabolic stress conditions (Michot et al. 2012; Zeharia et al. 2008). Moreover, lipin-1 deficiency disrupts phospholipid homeostasis and mitochondrial function, further exacerbating muscle pathology and systemic metabolic disturbances (Donkor et al. 2007). To date, the incidence of the disease does not appear to be clearly defined. The mutations result in episodes of acute, recurrent RML and exercise-induced myalgia, followed by symptoms such as muscle pain and weakness, and myoglobinuria (Indika et al. 2020). Episodes of RML are typically triggered by febrile illnesses but can also occur due to intense exercise, anesthesia, or fasting (Scalco et al. 2015; Michot et al. 2012). For this review, no studies were found that specifically highlight the outcomes of aerobic or anaerobic training protocols related to this disease. However, we identified two studies that evaluated the effects of exercise in individuals with lipin-1 deficiency, which may provide insights into the relationship between this disease and PE.

#### Exercise sessions

In 2018, Legendre et al. (2018) studied eight children (5 males, mean age 11.8) with confirmed lipin-1 mutations. The participants underwent CPET on a cycle ergometer until exhaustion to evaluate maximal exercise capacity. The assessments included cardiac output, stroke volume, ventricular mass index, and blood CK levels. CK levels were measured one hour before and then every 8 h for 24 h after the exercise test to monitor any signs of RML.

The study revealed a mismatch between oxygen delivery and utilization in muscle calculated as dQ/dVO₂, which represents the ratio between the change in cardiac output (Q) and oxygen consumption (VO₂). Notably, one patient performed the test twice, one of which was during a febrile state (38.5 °C). During the fever, post-exercise CK levels showed a significant increase (pre-test: 414 U/L; post-exercise: 8 h 1335 U/L, 16 h 2150 U/L, 24 h 6029 U/L), while under physiological temperature, this increment was significantly less pronounced (pre-test: 374 U/L; post-exercise: 8 h 460 U/L, 16 h 377 U/L, 24 h 477 U/L). This suggests that exercise performed under physiological conditions should not cause an excessive increase in CK levels. However, the combination of two factors that elevate metabolic demand, exercise and fever, poses a major RML risk.

#### Exercise sessions combined with a nutritional approach

Raaschou-Pedersen et al. (2019) investigated exercise tolerance and impaired FAO in a 48-year-old man with lipin-1 deficiency compared to two controls. The patient underwent submaximal tests, within a range corresponding to 59%–65% of the previously assessed V̇O₂max, on four occasions. After an initial preliminary session to measure total FAO and palmitate oxidation, the test was conducted with a glucose infusion at a concentration of 10%. The patient was then randomized to receive either a 2% glucose solution or a placebo solution (containing aspartame and acesulfame) before and during the test session. The patient demonstrated lower FAO and palmitate oxidation compared to controls, along with improved exercise capacity from the continuous glucose intake. A greater effect was observed during the infusion (exercise duration from 36 to 60 min) compared to oral supplementation (exercise duration from 36 to 46 min), while a slight decrement has been observed with the placebo solution (from 36 to 30 min). Results showed the highest level of CK after the first submaximal test (4400 U/L) and lower values when the exercises were preceded by IV glucose, oral placebo, and oral glucose intake (1,330 U/L, 676 U/L, and 752 U/L respectively). While the results suggest potential beneficial effects of infused and orally administered glucose, the marked reduction in CK levels observed in the placebo condition remains unexplained and underscores the need for further investigations.

## Discussion

In the present review, data from a total of 70 patients are included. For each study, information regarding patient characteristics, implemented interventions (training or exercise, with or without a nutritional approach), and the main outcomes are summarized in Table 1. A significant discrepancy is observed in the number of patients analyzed across different diseases: 40 with GSD V, 11 with VLCAD deficiency, 9 with Lipin-1 deficiency, 3 with GSD XIII, 2 with LCHAD deficiency and CPT II deficiency, and only 1 patient for GSD X, GSD XIV, and PGK1 deficiency. No cases are reported for GSD VII, GSD IXd, and GA II. Training programs have been evaluated in only three diseases: GSD V, CPT II deficiency, and VLCAD deficiency. Among these, both aerobic and anaerobic protocols have been assessed in GSD V, while only anaerobic protocols have been examined in CPT II deficiency and VLCAD deficiency. For all other conditions, except for GSD VII, GA II, and GSD IXd for which no study met the inclusion criteria, only studies assessing individual or few exercise sessions are available. In conditions where one or a few exercise sessions were evaluated, a CPET and/or a handgrip strength test were implemented. Overall, the present review highlights that, except for GSD V, there is a significant lack of data, and an urgent need for further research to identify exercise methodologies tailored to these disorders. Moreover, a major limitation of all these studies is their exclusive focus on PE, without considering the impact of daily physical activity, defined as any bodily movement that increases energy expenditure above resting levels (Caspersen et al. 1985). This aspect would certainly need further investigation into future studies.

**Table 1:** Summary of the characteristics of included studies

Disease	Author(s) and year	Subjects	Exercise/nutritional intervention and duration	Outcomes
GSD V	Haller et al. 2006	Eight patients (four males and four females) Mean age: 43.25 years (range: 33–61)	Aerobic training: cycling, 4 sessions per week at 60%–70% of maximal HR for a duration of 30 min per session during the first 7 weeks and of 40 min per session during the last 7 weeks, over a period of 14 weeks	↑ Peak cardiac output ↑ V̇O_2_max = CK
	Maté-Muñoz et al. 2007	Nine patients (four males, five females) Mean age: 36 ± 5 years (range: 17–69)	Aerobic training: walking or cycling, 5 sessions per week at 60% of peak HR for an incremental duration from 10 to 60 min per session, combined with a nutritional approach over a period of 8 months	↑ V̇O_2_peak ↑ Ventilatory threshold ↓ CK (Δ pre- and post-exercise, before and after the intervention): Pre-training: rest 2235.8 ± 2082.1 U/L, post-exercise 2283.8 ± 1875.6 Post-training: rest 595.1 ± 1074.3 U/L, post-exercise 711.4 ± 1104.0 U/L
	Lucia et al. 2007	A 29-year-old-female patient	Aerobic training: walking, 5 sessions per week at ~ 60% of maximal HR for an incremental duration from 10 to 60 min per session, combined with a nutritional approach over a period of 12 weeks	↑ V̇O_2_peak ↓ CK level (Δ pre- and post-exercise, before and after the intervention): Pre-training: rest 5278 U/L, post-exercise 5443 U/L Post-training: rest 448 U/L, post-exercise 460 U/L
	Pérez et al. 2007	A 38-year-old-male patient	Aerobic training: running, 3–4 sessions per week at ≤ 80%-85% of maximal HR for an incremental duration from 10 to 60 min per session, combined with a nutritional approach over a period of 7 months	↑ V̇O_2_peak ↓ CK (Δ pre- and post-exercise, before and after the intervention): Pre-training: rest 5682 U/L, post-exercise 5700 U/L Post-training: rest 2641 U/L, Post-exercise 2805 U/L
	Pérez et al. 2008	An 8-year-old-male patient	Aerobic training: 2–3 non-competitive swimming classes per week and 1–2 physical education classes per week, combined with a nutritional approach over a period of one year	↓ CK (Δ basal level pre- and post-intervention): From 1570 U/L to 251 U/L
	Pietrusz et al. 2018	Two patients, a 37-year-old male and a 46-year-old male	Anaerobic training: Patient 1, four sessions per week over a period of 4 years, composed by exercises mainly performed using free weights compound movements, with sets of 1–5 repetitions and a variable resting time of 2–5 min between sets and occasional sets of 4 repetitions with a rest of 30 s. Patient 2, protocol based on the use of resistance machines over a period of 3 months, with sets of 5 to 15 repetitions per exercise, stopping as soon as muscle discomfort occurred, and 1 min rest between sets	↑ Muscle strength for one patient ↑ Training workload for one patient = CK for one patient ↓ CK for one patient: From 2011 to 2014 CK mean: 3006 U/L From 2015 to 2017 CK mean: 1029 U/L 2017 July CK assessment: 941 U/L
	García-Benítez et al. 2013	A 15-year-old-male patient	Anaerobic training: 2 sessions of 60 min of 8 resistance exercises per week, with 2–3 sets of 10–15 repetitions at 60%–75% of 1RM, with 1–3 min rest between sets (after 5 min of cycle/rowing ergometer and 5 min of mobilization warm-up), combined with a nutritional approach over a period of 6 weeks	↑ Muscle power ↑ Muscle force ↓ CK: Post 1RM CK pre-training: 3636 U/L Post 1RM CK post-training: 1575 U/L
	Santalla et al. 2014	Seven patients (two males, five females) Mean age: 38.4 years (range 23–58)	Anaerobic training: 2 sessions of 4 resistance exercises performed as a circuit per week, with 4–6/6–8 sets of 5–6 repetitions at 6–7 of RPE, with 2–3 min rest between sets (after 12 min of arm crank ergometer plus 12 min of cycle ergometer warm-up), combined with a nutritional approach over a period of 16 weeks	↑ Lean mass ↑ Strength = CK levels
	Santalla et al. 2022	Ten patients (six males, four females) Mean age: 38 ± 18 years	Aerobic training: cycling, 5 sessions per week at 5–7 RPE and 0–1 RPP for an incremental duration from 15 to 60 min per session; anaerobic training: 2–3 sessions per week, with 3 circuit laps per session, each with 3 sets of 4 exercises with 6 repetitions at 6–7 OMNI-RPE and 0–1 RPP, with 2–3 min rest between sets, combined with a nutritional approach over a period of 2 years	↑ V̇O_2_peak ↑ Ventilatory threshold ↑ Training workload = CK level
GSD X	Kissel et al. 1985	A 24-year-old-male patient	Exercise sessions: running on treadmill during which speed and inclination were increased every minute, starting from 1.5 mph up to 5.5 mph and from a 4% inclination to 20%	= CK
GSD XIII	Buch et al. 2021	Three patients, a 21-year-old-male, a 41-year-old-male, and a 50-year-old-male	Exercise sessions: a hand grip strength test, a maximal incremental cycle ergometer test, and up to 1-h submaximal cycling exercise test at 65%–75% of V̇O_2_max	Glucose infusion = no effect ↑ CK from rest to peak: Patient 1, from 126 U/L (rest) to 374 U/L (peak) Patient 2, from 931 U/L (rest) to 2530 U/L (peak) Patient 3, from 94 U/L (rest) to 121 U/L (peak)
GSD XIV	Voermans et al. 2017	A 53-year-old-male patient	Exercise sessions: two incremental cycling tests peaked at 65 Watts and two constant submaximal 30 Watts-workload cycle ergometry, combined with a nutritional approach	↓ CK (Δ peak pre- and post-exercise, before and after the intervention): Pre-intervention: rest from 2500 U/L, post-exercise 13,600 U/L Post-intervention: rest from 875 U/L, post-exercise 3130 U/L
PGK-1	Hogrel et al. 2019	A 69-year-old-male patient	Exercise session: maximal hand grip strength test sustained for 30 s	↑ CK: From 544 U/L to 1920 U/L
CPT II	Parimbelli et al. 2021	A 14-year-old-female patient	Anaerobic training: 3 sessions of 1 h per week composed by an interval training performed as 1 min run at above 70% of maximal HR and 5 min walk, for an incremental duration from 15 to 30 min, followed by 6 resistance exercises with 3 sets of 8 repetitions at 6–7 of the OMNI scale, with 2 min rest between sets, combined with a nutritional approach over a period of 6 months	↑ V̇O_2_peak ↑ Peak power output ↑ Ventilatory threshold ↑ Oxygen pulse = CK
	Crisafulli et al. 2024a	An 18-year-old-male patient	Anaerobic training: the patient kept his competitive sport activity but no specifics are available, combined with a nutritional approach over a period of 5 months	= CK
LCHAD	Karall et al. 2014	A 13-year-old-male patient	Exercise sessions: incremental cycle test, with increasing of 25 Watts every 2 min until exhaustion, and 3 endurance tests of 30 min each at 80, 90, and 100 Watts, respectively, combined with a nutritional approach	= CK
	van Eerd et al. 2017	A 34-year-old-female patient	Exercise sessions: 4 min of unloaded cycling warm-up followed by increasing workload of 1 Watt every 5 s at a pedal frequency of 60–80 RPM until exhaustion, combined with a nutritional approach over a period of one month	↑ Peak power output ↑ HR ↑ V̇O_2_max ↓ CK (rest and max): Session 1: rest 183 IU/L, max 197 IU/L Session 2: rest 135 IU/L, max 139 IU/L Session 3: rest 110 IU/L, max 115 IU/L
VLCADD	Herrera-Olivares et al. 2019	A 23-year-old-female patient	Anaerobic training: one-month of 2 HIIT sessions per week followed by 5 months of 2 HIIT sessions plus 2 resistance exercises sessions per week (HIIT sessions consisted of 6 sets of 70–80 s on a cycle ergometer at a pedal frequency of minimum 100 RPM with 1 min rest between sets. The 6 resistance exercises were performed as 2 circuit laps of 4–7 repetitions at 5–7 of OMNI scale with 2 min rest between sets and 4 min rest between laps), combined with a nutritional approach over a period of 6 months	↑ V̇O_2_peak ↑ Maximal power output ↑ Maximal HR = CK
	Diekman et al., 2016	Five patients (four males and a female) Mean age: 28 years (range: 13–37)	Exercise sessions: maximal incremental cycle ergometer test with increasing of 35 Watts every 3 min until exhaustion, and endurance cycle ergometer test at FATMAX for a duration of 45 min	= CK in 3 patients ↑ CK in 2 patients of which one RML: Patient 1: from 156 U/L to 576 U/L Patient 2 (RML): from 36 U/L and to 400 U/L
	Bleeker et al., 2020	Five patients (four males and a female) Median age: 22 years (range: 17–45)	Exercise sessions: maximal incremental cycle ergometer test and endurance cycle ergometer test at FATMAX at a pedal frequency of 60–80 RPM for a duration of 45 min, combined with a nutritional approach	↑ HR = CK
LPIN-1	Legendre et al. 2018	Eight patients (five males and three females) Mean age: 11.8 ± 3.6 years (range: 6.1–15.5)	Exercise session: maximal incremental cycle ergometer test at increasing load from 5 to 15 Watts until exhaustion)	= CK
	Raaschou-Pedersen et al. 2019	A 48-year-old-male patient	Exercise sessions: a maximal exercise cycle ergometer test to exhaustion and 4 one-hour submaximal cycle ergometer exercise tests at ~ 60% of V̇O_2_max, combined with a nutritional approach	Less post-exercise CK increments with IV glucose, oral placebo, and oral glucose compared to a preliminary session Post-preliminary session CK: 4400 U/L Post-IV glucose session CK: 1330 U/L Post-placebo session CK: 676 U/L Post-oral glucose session CK: 752 U/L

↑, increased; ↓, decreased; = equality

BPM, beats per minute; CK, creatine kinase; CPT II, Carnitine Palmitoyl-Transferase II; FATMAX, maximal fat oxidation; HR, heart rate; HIIT, high-intensity interval training; IU/L, international unit per liter; IV, intravenous; GSD, glycogen storage disease; 1RM, one-repetition maximum; LCHAD, long-chain 3-hydroxyacyl-CoA dehydrogenase; LPIN1, lipin-1; mph, miles per hour; PGK-1, phosphoglycerate kinase-1; RPE, rating of perceived exertion; RPP, rating of perceived pain; U/L, unit per liter; VLCADD, very-long-chain acyl-CoA dehydrogenase deficiency; V̇O_2_max, maximal oxygen uptake; V̇O_2_peak, peak oxygen uptake

Nonetheless, although limited, the available evidence offers some insights that may serve as a valuable theoretical foundation for future validation studies.

In GSD V, the reported data indicates that both aerobic and anaerobic training are feasible when accompanied by a tailored nutritional strategy, in line with current guideline recommendations (Lucia et al. 2021). Due to their inability to utilize muscle glycogen for energy, GSD V patients experience an energy mismatch during the initial stages of exercise, which increases their susceptibility to exercise-induced RML. Based on this understanding, a well-structured dietary approach plays a protective role in both aerobics (Maté-Muñoz et al. 2007; Lucia et al. 2007; Pérez et al. 2007, 2008) and anaerobic training (García-Benítez et al. 2013; Santalla et al. 2014, 2022). In all studies where training was combined with nutrition, participants consumed simple carbohydrates (such as glucose and fructose) shortly before exercise, along with complex carbohydrates (like pasta, rice, or bread) in meals preceding the training sessions. Simple carbohydrates provide patients with a rapidly absorbable and immediately available energy source, while complex carbohydrates help maintain optimal liver glycogen stores. This approach ensures a continuous glucose supply during the early phases of exercise, enabling patients to counteract the early onset of severe exercise intolerance (Salazar-Martínez et al. 2021) and support the second wind phase, wherein the body relies on extra-muscular energy sources (i.e., plasma glucose derived from hepatic glycogen reserves) (Haller and Vissing 2002). The protective effect of glucose availability is further supported by Haller et al. (2006), who found that despite the absence of a specific nutritional program accompanying the training, intravenous glucose infusion allowed patients to exercise without an increase in CK levels. Notably, aerobic training has been associated with improvements in aerobic capacity and physical efficiency, as well as a reduced susceptibility to exercise-induced RML, evidenced by post-training decreased CK levels (Maté-Muñoz et al. 2007; Lucia et al. 2007; Pérez et al. 2007, 2008). In this regard, the effects of anaerobic training appear to be less pronounced, although present. Notably, one study reported a post-training reduction in CK levels in a single patient (Pietrusz et al. 2018), while another observed a smaller increase in CK levels following the post-training maximal 1RM test compared to the pre-training test (García-Benítez et al. 2013). The physiological processes underlying the reduction in CK levels are not yet fully understood. However, the majorly proposed hypothesis is that the stimulus for muscle growth and protein synthesis prompted by exercise may partially counterbalance the increased susceptibility to muscle injury and muscle wasting characteristic of the disease (Lucia et al. 2007; Maté-Muñoz et al. 2007; Pérez et al. 2007, 2008). Similarly, Pietrusz et al. (2018) further suggest that strength training may increase muscle fiber resistance to damage. Although this protective effect of exercise is now well recognized and has been incorporated into recent clinical guidelines (Lucia et al. 2021), the molecular mechanisms underlying such adaptations have yet to be fully clarified. GSD XIV shares several characteristics with GSD V, such as early-onset exercise intolerance and the second wind phenomenon (Preisler et al. 2017; Salazar-Martínez et al. 2021). Given these similarities, the observed post-exercise reduction in CK levels following galactose supplementation (Voermans et al. 2017), a carbohydrate routinely recommended for GSD XIV as an alternative to glucose (Altassan et al. 2021), aligns with findings in GSD V, where pre-exercise glucose intake is consistently reported across all analyzed supplementation studies and is recommended in clinical guidelines. This suggests that the underlying mechanisms contributing to improved exercise tolerance and reduced muscle damage may be similar across these conditions. The rationale behind these observations likely stems from the ability of specific carbohydrates to provide readily available energy sources during exercise, thereby mitigating the risk of exercise-induced complications before the onset of the second wind. Data from exercise sessions in other GSDs provide insights that may help define future research objectives. Analysis of CK levels has consistently shown a variable post-exercise increase across all conditions (Kissel et al. 1985; Buch et al. 2021; Hogrel et al. 2019), with one case reporting exercise-induced RML (Voermans et al. 2017). For instance, in PGK-1 deficiency, the only available case report documented a significant increase in CK following isometric exercise at 70% of maximal handgrip strength (Hogrel et al. 2019). This finding highlights the need for caution when recommending anaerobic exercise for these patients. In GSD X, a case report indicated a modest CK elevation two minutes post-exercise (Kissel et al. 1985); however, this timing may not be ideal for assessing the risk of RML, as CK fluctuations often occur at a greater temporal distance from the end of an exercise session. This has been observed in several studies that suggest CK levels can peak later after exertion (Giannoglou et al. 2007; Crisafulli et al. 2024b; Bäcker et al. 2023). Unfortunately, no differences in CK variations following aerobic versus anaerobic exercise have been reported in any GSD, except for GSD V. This lack of distinction complicates the identification of the safest type of exercise for these patients. Furthermore, a combined approach integrating training and nutritional interventions has not yet been explored in GSD X, GSD XIII, and PGK-1 deficiency. Given the benefits observed in other GSDs such as GSD V and GSD XIV, further investigation into this strategy might reveal valuable. Exploring how tailored nutritional strategies can complement exercise training could lead to improved outcomes and safety for patients with these conditions.

The number of studies on training interventions for FAO disorders meeting our inclusion criteria is significantly lower compared to those on GSDs. To date, only two case reports on CPT II deficiency (Parimbelli et al. 2021; Crisafulli et al. 2024a) and one on VLCAD deficiency (Herrera-Olivares et al. 2019) are available. However, despite the limited data, the findings appear consistent, suggesting that anaerobic, high-intensity, short-duration exercise may be a feasible approach for these conditions. Importantly, the use of a high-carbohydrate, low-fat diet, along with MCTs supplementation is a common trait of all three studies, suggesting the potential role that such nutritional strategy may play in making such training modalities sustainable by the patients. The importance of appropriate dietary support is also highlighted by studies on exercise sessions, where supplementation with simple carbohydrates, MCTs and ketone esters enabled patients to sustain CPET sessions (Karall et al. 2014; van Eerd et al. 2017; Bleeker et al. 2020; Raaschou-Pedersen et al. 2019). Specifically, supplementation with MCTs seems beneficial for supporting energy metabolism in disorders characterized by impaired enzymatic activity that compromises, at various stages, the capacity to oxidize LCFAs (Crisafulli et al. 2024a, Parimbelli et al. 2021; Herrera-Olivares et al. 2019; van Eerd et al. 2017). This might be explained by the fact that MCTs enter mitochondria more readily (Pereyra et al. 2023), bypassing such enzymatic deficits and providing a lipid-based substrate that can be efficiently utilized for energy production. Similarly, ketone bodies, as water-soluble molecules, do not require the enzymatic machinery necessary for mitochondrial transport of LCFAs (Evans et al. 2017) and may exert beneficial effects in these patients, as proposed by Bleeker et al. (2020). In contrast, in studies where the exercise sessions were not accompanied by specific nutritional supplementation, significant increases in CK levels were observed (Diekman et al. 2016; Legendre et al. 2018). Interestingly, MCT supplementation has been proposed not only to sustain energy availability during PE but also to support metabolic recovery in the post-exercise phase. In fact, following high-intensity exercise, Excess Post-Exercise Oxygen Consumption (EPOC) may occur, leading to elevated FAO (Wolfe et al. 1990). Theoretically, in individuals with impaired FAO, this shift may represent a potential metabolic stressor. Notably, Herrera-Olivares et al. (2019), who proposed high-intensity training in a patient with VLCAD deficiency, have suggested that pre-exercise MCT supplementation may serve as a preventive countermeasure by providing an alternative lipid substrate that can be safely oxidized, potentially mitigating post-exercise metabolic stress. This hypothesis could also explain the absence of adverse events reported in the context of CPT II deficiency by Parimbelli et al. (2021) and Crisafulli et al. (2024a), who similarly implemented high-intensity exercise protocols and included MCT supplementation in the nutritional plan. However, whether high-intensity exercise leads to a sufficiently elevated post-exercise fatty acid demand to induce clinically relevant metabolic stress in these patient populations remains a hypothesis that requires further investigation.

### Possible elements for exercise adaptation

**Fig. 2:**
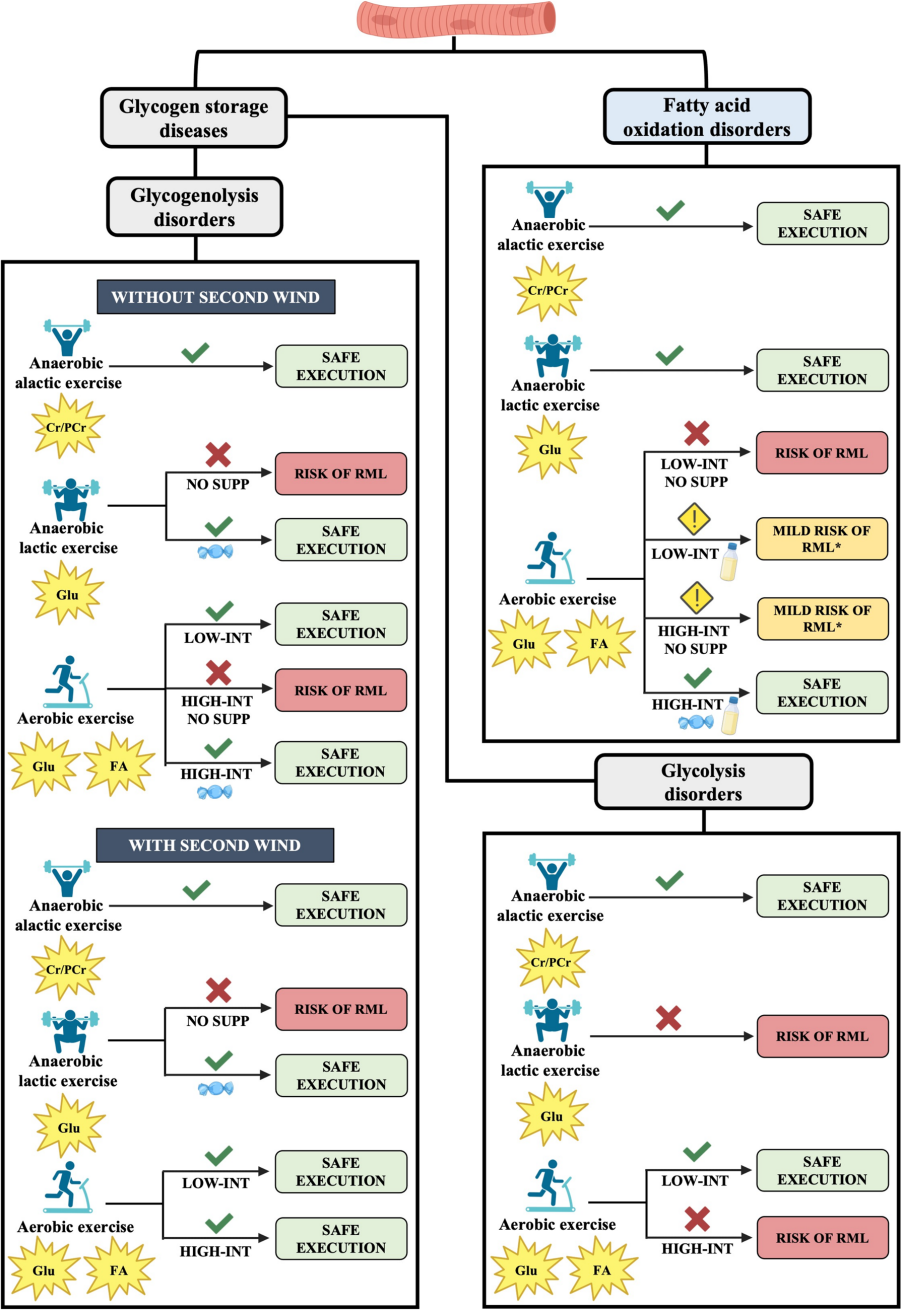
Graphical representation of the proposed theoretical principles of exercise adaptation. In GSDs, we suggest distinguishing exercise adaptation principles between disorders of muscular glycolysis and those of glycogenolysis, and further differentiating glycogenolysis disorders based on the presence or absence of the second wind phenomenon. In all these subcategories, anaerobic alactic exercise, which relies on phosphagens as energy substrates, might be performed safely. Nevertheless, future research may explore whether Cr supplementation might be beneficial in GSDs for enhancing the ability to perform repeated bouts of short-duration, high-intensity exercise. In fact, it is well known that repeating such activities leads to a rapid depletion of PCr and a concomitant increase in glycolytic activity (Gaitanos et al., 1993). Therefore, Cr supplementation, by increasing muscular Cr concentration and enhancing PCr resynthesis (Greenhaff et al. 1994), could potentially increase tolerance to these activities by delaying reliance on carbohydrate metabolism, thus prolonging safety during such exercise performance

Although, apart from GSD V, the available data are limited, interesting elements for exercise adaptation in the context of metabolic myopathies seem to emerge from the analysis. As previously discussed, RML would be triggered by a mismatch between energy demand and supply (Ørngreen and Vissing 2017; Chavez et al. 2016). Therefore, the critical factor in preventing RML would be to avoid conditions where the muscle is unable to access adequate substrates to meet the increased metabolic demands induced by exercise. In this light, two key factors could provide a sensible basis for exercise adaptation. First, the identification of which energy substrate utilization is impaired by the disease. Second, based on the well-established notion that phosphagens are the primary energy substrate in anaerobic alactic activity, carbohydrates in anaerobic lactic activity, and carbohydrates/fats in aerobic exercise (Hargreaves and Spriet 2020), selecting an exercise modality that involves the utilization of an energy substrate that is metabolically available to the patient. Furthermore, ensuring the availability of the appropriate energy substrate through tailored nutritional strategies and/or supplementation becomes an additional pivotal factor. This would be supported by the fact that in diseases where there is an inability to consume glycogen, such as in GSD V and GSD XIV, simple carbohydrates administration (glucose and galactose, respectively) has proven to be an effective strategy to sustain exercise phases prior to the second wind phenomenon while, in later phases, no limitations have been reported, possibly due to the availability of alternative energy sources. Consistently, the implementation of training based on bouts of no more than 10 s, relying mainly on phosphagen metabolism, has been proposed as safe and feasible in GSD V, even in the absence of specific nutritional interventions (Pietrusz et al. 2018; Lucia et al. 2021). This perspective appears to be in line with findings observed in another GSD (PGK1 deficiency), where a handgrip strength test sustained for approximately 30 s, an anaerobic lactic performance, resulted in a more than threefold increase in CK levels compared to baseline (Hogrel et al. 2019). In contrast, more modest increases were reported following a maximal exercise test on a cycle ergometer by Butch et al. (2021) in three GSD XIII patients, possibly due to the nature of the test, which involves both anaerobic and aerobic phases and, consequently, the utilization of both carbohydrates and fatty acids. This rationale would also explain the observed feasibility of short-duration, high-intensity exercises in FAO disorders, such as CPT II deficiency (Parimbelli et al. 2021; Crisafulli et al. 2024a) and VLCAD deficiency (Herrera-Olivares et al. 2019). Furthermore, it aligns with the observation that, in FAO impairments, maximal exercise tests involving an aerobic component, when accompanied by supplementation with utilizable substrates related to aerobic metabolism (carbohydrates and MCTs), did not result in significant peri-exercise (van Eerd et al. 2017) or post-exercise CK elevations (Karall et al. 2014; Bleeker et al. 2020; Raaschou-Pedersen et al. 2019). In contrast, tests performed without supplementation led to RML in a VLCAD deficiency patient (Diekman et al. 2016). These considerations remain theoretical at this stage but may provide a valuable rationale for designing future studies on PE adaptation in metabolic myopathies. A graphical representation of the proposed theoretical approach is available in Fig. 2. For a clearer interpretation of the figure, it is recommended to consult the caption.

Anaerobic lactic exercise, primarily deriving energy from carbohydrates, may induce RML in all three subgroups. However, while such risk may be unavoidable in glycolysis disorders, in those affecting glycogenolysis, regardless of the presence of the second wind phenomenon, supplementation with simple sugars (such as glucose or fructose) may allow safe execution. Importantly, aerobic exercise should be considered in relation to its intensity, since it is well established that, at low intensity, it primarily relies on fat oxidation, whereas increasing intensity shifts the energy demand mainly toward carbohydrate metabolism (Venables et al. 2005). Therefore, in the absence of supplementation, low-intensity aerobic exercise might be safely performed across all three subcategories, while high-intensity aerobic exercise may pose an unavoidable risk of RML in glycolytic disorders, and a potentially avoidable one, through simple sugar supplementation, in glycogenolysis disorders without second wind. Moreover, both at low and submaximal intensities, aerobic exercise would be safely feasible in glycogenolysis disorders with the second wind, once the phenomenon has been achieved. About this latter category, it is crucial to underline that the attainment of the second wind represents a key condition for safe exercise, since initiating high-intensity training prior to this metabolic shift may result in RML and myoglobinuria.

In FAO disorders, both anaerobic alactic and lactic exercises, relying on phosphagens and carbohydrates, might be performed safely. During aerobic exercise, the prolonged maintenance of low intensity may pose a risk of RML. Although MCTs, which are commonly advised in FAO disorders, may partially mitigate this risk providing a lipidic energetic substrate, it is the authors’ opinion that low-intensity aerobic activity remains inadvisable in these patients. In contrast, high-intensity aerobic exercise, when accompanied by appropriate supplementation with MCTs and carbohydrates, may be safer. Notably, MCT supplementation could play a particularly important role in supporting the initial phases of aerobic metabolism, before the energy substrate shifts toward carbohydrates (Venables et al. 2005), whereas engaging in high-intensity aerobic activity without adequate supplementation, while still relying to some extent on lipid metabolism, could expose the patient to an increased risk of RML. Based on this rationale, it is possible that ketone supplementation may also have a similar protective role. In this context, carbohydrate supplementation may be useful in providing an alternative energy source to compensate for the reduced use of fats.

Importantly, it should be noted that the available data regarding nutritional and PE interventions analyzed in this work and forming the theoretical rationale illustrated in the figure derive primarily from short-term studies conducted under controlled experimental conditions. While these strategies may offer acute benefits in terms of exercise tolerance, their long-term safety remains uncertain. In particular, sustained high intake of simple sugars such as sucrose has been associated with increased metabolic and cardiovascular risks (Ma et al. 2022). Such short-term findings cannot be proposed as long-term clinical recommendations. This underscores the need for longitudinal studies to better elucidate the chronic effects of such interventions and to support the development of evidence-based, individualized guidelines.

Moreover, it is important to acknowledge that the representation presented herein is necessarily schematic, intended to enhance conceptual clarity. Individual tolerance to various energy metabolism pathways may be modulated by the degree of residual enzymatic activity, which can vary substantially even among patients affected by the same disorder (Bhai 2021). For instance, in FAO disorders a certain degree of residual enzymatic activity may allow for some tolerance to low-intensity aerobic activity, especially if not particularly prolonged and, consequently, not involving a high amount of FAO.

Cr, creatine; FA, fatty acid; Glu, glucose; INT, intensity; MCTs, medium-chain triglycerides; PCr, phosphocreatine; RML, rhabdomyolysis; SUPP, supplementation; , simple sugars;, MCTs.

## Conclusions

The literature reviewed indicates a significant knowledge gap regarding the feasibility and safety of PE in metabolic myopathies that are at risk for RML. The most substantial evidence is available for GSD V, while data on other conditions are primarily limited to small patient cohorts or case reports. This underscores the urgent need for further research, as developing disease-specific exercise protocols could greatly enhance clinical management and improve patients' quality of life. An emerging principle suggests that exercise modalities should be tailored to each patient's residual metabolic capacity. Coupled with a nutritional strategy to ensure that muscles can meet the metabolic demands imposed by exercise, this approach represents a promising direction for future studies.

## Data Availability

All information supporting the findings of this study are available within the paper.

## Abbreviations

ACADVL: Very long-chain acyl-CoA dehydrogenase
ATP: Adenosine triphosphate
BMI: Body mass index
Ca^2^⁺: Calcium
CHO: Carbohydrates
CK: Creatine kinase
CPET: Cardiopulmonary exercise testing
CPT II: Carnitine palmitoyl-transferase II
Cr: Creatine
DEXA: Dual energy x-ray absorptiometry
ENO3: Enolase 3
ET-QO: ETF-ubiquinone oxidoreductase
ETF: Electron transfer flavoprotein
FA: Fatty acid
FAO: Fatty acid oxidation
FATMAX: Maximal fat oxidation
g: Gram
GA II: Glutaric aciduria type II
Glu: Glucose
GSD: Glycogen storage disease
HADHA: Hydroxyacyl-CoA dehydrogenase trifunctional multienzyme complex subunit alpha
HIIT: High-intensity interval training
HR: Heart rate
Int: Intensity
IU/L: International unit per liter
IV: Intravenous
K⁺: Potassium
kcal: Kilocalories
KE: Ketone esters
kg: Kilogram
LCFA: Long-chain fatty acids
LCHAD: Long-chain 3-hydroxyacyl-CoA dehydrogenase
LPIN1: Lipin-1
MCT: Medium-chain triglycerides
mg: Milligram
ml: Milliliter
mM: Millimolar
MR: Magnetic resonance
Na⁺: Sodium
PCr: Phosphocreatine
PE: Physical exercise
PFK: Phosphofructokinase
PFKM: Muscle phosphofructokinase
PGAM2: Phosphoglycerate mutase 2
PGK: Phosphoglycerate kinase
PGK-1: Phosphoglycerate kinase-1
PGM1: Phosphoglucomutase 1
PhK: Phosphorylase kinase
Pi: Inorganic phosphate
PYGM: Glycogen myophosphorylase
Q: Cardiac output
QoL: Quality of life
RER: Respiratory exchange ratio
RM: Repetition maximum
RML: Rhabdomyolysis
RPE: Rating of perceived exertion
Supp: Supplementation
TFP: Mitochondrial trifunctional protein
U/L: Unit per liter
VLCAD: Very-long-chain acyl-CoA dehydrogenase
V̇O_2_: Oxygen consumption
V̇O₂max: Maximal oxygen uptake
V̇O₂peak: Peak oxygen uptake
